# Dapagliflozin treatment of patients with chronic kidney disease without diabetes across different albuminuria levels (OPTIMISE-CKD)

**DOI:** 10.1093/ckj/sfae100

**Published:** 2024-04-04

**Authors:** Maria K Svensson, Navdeep Tangri, Johan Bodegård, Samuel Adamsson Eryd, Marcus Thuresson, Tadashi Sofue

**Affiliations:** Department of Medical Sciences, Renal Medicine, Uppsala University, Uppsala, Sweden; Uppsala Clinical Research Centre, Uppsala, Sweden; University of Manitoba Max Rady College of Medicine, Winnipeg, MB, Canada; Cardiovascular, Renal and Metabolism Evidence, BioPharmaceuticals Medical, AstraZeneca, Gothenburg, Sweden; Cardiovascular, Renal and Metabolism Evidence, BioPharmaceuticals Medical, AstraZeneca, Gothenburg, Sweden; Statisticon AB, Uppsala, Sweden; Department of Cardiorenal and Cerebrovascular Medicine, Kagawa University, Takamatsu, Kagawa, Japan

**Keywords:** albuminuria levels, chronic kidney disease, clinical outcomes, dapagliflozin, eGFR

## Abstract

**Background:**

We compared kidney and cardiorenal protection in patients without type 2 diabetes across urine albumin–creatinine ratio (UACR) levels after initiation on dapagliflozin for the treatment of chronic kidney disease (CKD).

**Methods:**

OPTIMISE-CKD is an observational study describing dapagliflozin treatment for CKD. Adult patients with CKD without type 2 diabetes were included in the primary analysis. Baseline UACR was grouped as normal/mildly elevated (0–29 mg/g), low (30–200 mg/g) and high (>200 mg/g). Outcomes were estimated glomerular filtration rate (eGFR) trajectories/slopes, cardiorenal complications and all-cause mortality.

**Results:**

In total, 1480 patients had low (*n* = 796) and high (*n* = 684) UACR. The two groups were similar at baseline, aged 75 and 74 years, and 42% and 39% female, respectively. After dapagliflozin initiation, an acute eGFR dip of 3 mL/min/1.73 m^2^ was observed, followed by a flat development in both groups. The eGFR slope [95% confidence interval (CI)] for patients with low UACR was 0.79 mL/min/1.73 m^2^ per year (–0.59, 2.56), and similar to patients with high UACR [0.40 mL/min/1.73 m^2^ per year (–0.46, 1.38)]. Risks of cardiorenal complications and all-cause mortality were similar, with adjusted hazard ratios of 0.89 (95% CI 0.66, 1.19) and 1.10 (95% CI 0.63, 1.92), respectively. Analogous results were found in those with normal/mildly elevated UACR.

**Conclusions:**

Dapagliflozin in patients without type 2 diabetes for the treatment of CKD demonstrated similar kidney protection, cardiorenal and all-cause mortality risk across UACR levels. This suggests that the efficacy of dapagliflozin found in clinical trials expands to real-world patients with CKD, regardless of albuminuria levels.

## Graphical Abstract

**Graphical Abstract:**
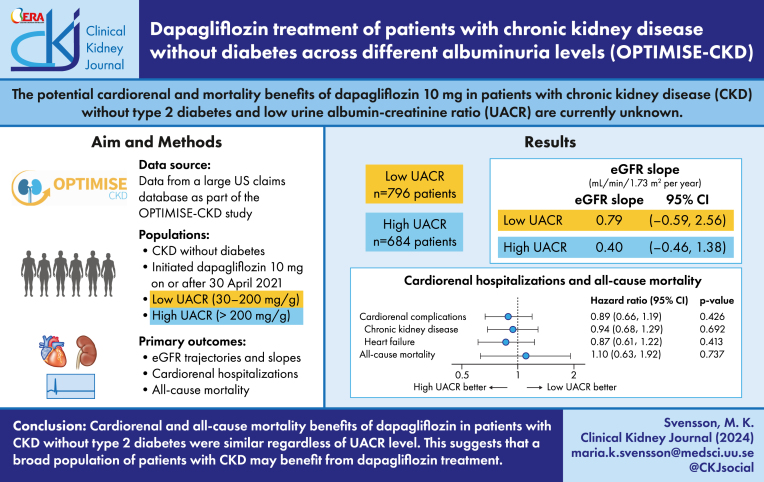

KEY LEARNING POINTS **What was known:**

- Renin–angiotensin system inhibitors had been the mainstay treatment of chronic kidney disease (CKD) for decades until clinical trials showed that sodium–glucose co-transporter-2 inhibitors (SGLT2i) have paradigm-shifting efficacy on kidney protection and mortality.
- Although the renal benefits of SGLT2i have been extensively reported, evidence for patients with lower urine albumin–creatinine ratio (UACR) levels (<200 mg/g), particularly in those without diabetes, is limited.
- New clinical trials to explore efficacy in patients with CKD without type 2 diabetes and low UACR are not expected within the foreseeable future, potentially leaving these patients without access to the SGLT2i class; results from a real-world clinical setting can be used to understand the SGLT2i effects in this patient group.

**This study adds:**

- This is one of the first real-world studies to understand the novel use of dapagliflozin (the first SGLT2i approved for CKD treatment, regardless of presence of type 2 diabetes) in the treatment of patients with CKD without type 2 diabetes across different UACR levels.
- The study shows that the kidney-protective effects demonstrated in clinical trials expand to patients with CKD with lower UACR levels.
- The study also shows that hard outcomes, like CKD hospitalizations, heart failure hospitalizations and all-cause mortality, seem to be equally prevented across all UACR levels.

**Potential impact:**

- The results from the present study suggest that dapagliflozin has equipotent real-world effectiveness regardless of UACR status in patients with CKD without type 2 diabetes.
- These findings add to a growing body of evidence from real-world clinical use, suggesting that dapagliflozin is effective in a broad CKD population.

## INTRODUCTION

Chronic kidney disease (CKD) is one of the most prevalent non-communicable diseases worldwide, currently estimated at 1 in 10 people, or 800 million people in total [1–4]. Patients with CKD are at high risk of severe cardiorenal complications, such as hospitalizations for CKD and heart failure, which places a huge burden on healthcare systems [1, 5, 6]. Importantly, cardiorenal complications have been reported to be a greater clinical problem in patients with CKD compared with atherosclerotic cardiovascular diseases [1, 7]. Recently, paradigm-shifting clinical trials in patients with CKD have shown that sodium–glucose co-transporter-2 inhibitors (SGLT2i) reduce the risk of cardiorenal complications, CKD progression, heart failure hospitalizations and death, regardless of diabetes status [8–13]. Indeed, the KDIGO clinical practice guidelines now recommend that CKD be treated with a SGLT2i alongside a renin–angiotensin system inhibitor (RASi) [14]. However, clinical trials showing positive kidney-protective efficacy in patients with CKD without type 2 diabetes included only patients with high urine albumin–creatinine ratio (UACR; >200 mg/g) [11, 15], resulting in little evidence for patients with low UACR [16]. The threshold of UACR 200 mg/g used in clinical trials [11, 15], as compared with 300 mg/g for the KDIGO classification [14], was driven by the anticipated balance between number of patients and potential number of events in the trials’ power calculations.

Dapagliflozin was the first SGLT2i to be approved for use in patients with CKD, with or without type 2 diabetes [11, 17, 18], and has been studied in a real-world clinical setting [7, 19]. Although the beneficial effects of dapagliflozin in patients with CKD with type 2 diabetes and low UACR have been shown [20], effects in patients with CKD without type 2 diabetes and with low UACR have been little studied [16, 19]. Despite its broad indication, uptake of dapagliflozin for CKD among patients without type 2 diabetes with low UACR may be hindered by the limited number of studies performed in this group [7].

The current analysis used contemporary real-world clinical data extracted from a well-established claims database in the USA. The aim was to compare estimated glomerular filtration rate (eGFR) trajectories, eGFR slopes and cardiorenal and all-cause mortality outcomes of dapagliflozin 10 mg in patients with CKD without type 2 diabetes, with low (30–200 mg/g) versus high (>200 mg/g) UACR.

## MATERIALS AND METHODS

### Study design

This study is part of the OPTIMISE-CKD study [7], and used data from the USA (Supplementary Methods). The primary analysis included patients with CKD without type 2 diabetes; supportive analysis included patients with CKD with type 2 diabetes.

### Study populations and study periods

Patients aged 18 years or older were included if they met the CKD definition at any time during the study period (Supplementary data, Table S1). CKD was defined as having either two eGFR measurements ≤60 mL/min/1.73 m^2^ taken ≥90 days apart or a first eGFR ≤60 mL/min/1.73 m^2^ followed by a first CKD diagnosis at any time, including chronic, acute, hypertensive, diabetic, tubular and glomerular renal disease prior to the index date (Supplementary data, Table S2). Patients with prior use of SGLT2i, CKD stage 5 (based on eGFR <15 mL/min/1.73 m^2^ or dialysis) or type 1 or gestational diabetes were excluded.

### Index date

**Figure 1:**
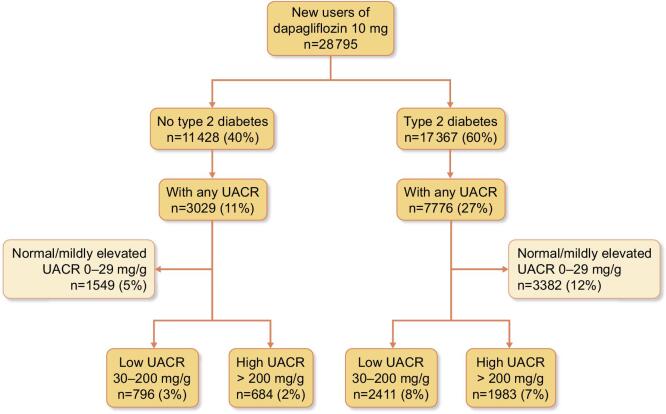
New users of dapagliflozin 10 mg in patients with CKD in the USA in the years 2021–23 (post-approval).

Patients with CKD were indexed at the date of new initiation of dapagliflozin 10 mg between 30 April 2021 (date of CKD approval for dapagliflozin) and 31 March 2023 (date of data extraction; Fig. 1).

### UACR groups

Patients were grouped according to baseline UACR: ‘low UACR’ was defined as 30–200 mg/g and ‘high UACR’ defined as >200 mg/g. The 200 mg/g threshold was chosen based on high UACR definition and inclusion criterion in clinical trials [11, 15]. Patients with normal/mildly elevated UACR (0–29 mg/g) were also assessed.

### Patient characteristics

Characteristics were described prior to index, including demographics, comorbidities and treatments (Supplementary data, Tables S2, S3 and S4). Use of treatments was based on at least one filled prescription during the year prior to the index date.

### Ethical approval

Data sources used in this project are subject to ethical and privacy restrictions. See Supplementary Appendix for details.

### eGFR outcomes

The absolute difference in eGFR relative to baseline was described at 3 and 6 months prior to index and 1, 3, 6, 9 and 12 months after index. Each time point was described by the closest value relative to the specific time point with a window of ±1.5 months, except for the 1-month after-index value, which had a window of ±1 month. Consequently, the value for 3 months post-baseline included values from 2 to 4.5 months.

Individual eGFR slopes were calculated using linear regression models based on all post-baseline measurements. To contribute to the slope, at least two measurements had to be available, with at least 30 days between the first and last measurement.

### Clinical outcomes

The following clinical outcomes were described for each patient during the 12 months after index date: in-patient hospitalizations with a diagnosis of CKD (including diagnoses of acute kidney failure, unspecified kidney failure, diabetic kidney disease, hypertensive CKD, dialysis, glomerular diseases, renal tubulointerstitial disease or other), heart failure and all-cause mortality (Supplementary data, Table S2). Events were determined using both a broad and a strict definition of cardiorenal complications. The broad definition included patients with a diagnosis of cardiorenal complication (CKD or heart failure) in an in-hospital setting. The strict definition was restricted to patients with a hospital admission where a cardiorenal complication was the main diagnosis.

### Statistical analysis

All analyses were performed in the low and high UACR groups. Baseline characteristics were described using interquartile ranges (IQR) for continuous variables and frequencies (%) for categorical variables. Change in eGFR relative to baseline was described as the mean change at each time point with 95% confidence intervals (CI) based on observed values. The individual patient slopes of post-eGFR measurements were analysed using quantile regression, where the median slopes (per year) were estimated and presented with 95% CI. Three models were used: unadjusted, eGFR adjusted (adjusted by baseline eGFR) and multivariable adjusted [baseline eGFR, age, sex, heart failure and RASi (including angiotensin receptor–neprilysin inhibitors)]. The time to clinical outcomes was analysed using Cox regression models, where time since index was the primary timescale. The models were adjusted for age, sex, heart failure, CKD diagnosis, myocardial infarction, stroke and peripheral arterial disease. The results were presented as the hazard ratio with 95% CI for the relative risk of high UACR relative to low UACR. The crude event rates were presented as number of events per 100 patient-years. In all analyses, the primary cohorts were patients with CKD without type 2 diabetes, grouped by low and high UACR. All analyses have also been performed within patients with CKD and with type 2 diabetes.

## RESULTS

In total, 28 795 new users of dapagliflozin 10 mg were identified after its approval for CKD on 30 April 2021 (Fig. 1). In patients without type 2 diabetes, 3029 (27%) had an UACR reading, of whom 796 (26%) had low, 684 (23%) had high and 1549 (51%) had normal/mildly elevated UACR. In patients with type 2 diabetes, 7776 (45%) had an UACR reading, of whom 2411 (31%) had low, 1983 (26%) had high and 3382 (43%) had normal/mildly elevated UACR.

### Baseline characteristics

Dapagliflozin initiators with low UACR were slightly older, had more comorbidities (myocardial infarction, atrial fibrillation and heart failure) and were less frequently treated with RASi compared with those with high UACR (Table 1). The majority of patients had a registered diagnosis of hypertensive CKD (55%–68%) followed by tubular kidney disease (13%–17%), glomerular kidney disease (4%–11%) and renal arterial stenosis (4%–5%). Patients with normal/mildly elevated UACR were similar to those with low UACR (Supplementary data, Table S5). Corresponding baseline characteristics in low and high UACR for patients with type 2 diabetes were comparable, although the differences were less pronounced (Supplementary data, Table S6).

**Table 1:** Baseline characteristics: patients with CKD without type 2 diabetes newly initiated on dapagliflozin 10 mg in the USA in the years 2021–23 (post-approval).

	Patients without type 2 diabetes	
	Low UACR 30–200 mg/g	High UACR >200 mg/g
Number of patients, *n* (%)	796 (26)	684 (23)
Age, years, mean (SD)	75 (8)	74 (9)
Female, *n* (%)	336 (42)	264 (39)
Days since 1st CKD diagnosis, median (IQR)	1347 (618–2024)	1169 (538–2067)
Comorbidities, *n* (%)		
Atherosclerotic cardiovascular disease		
Myocardial infarction	215 (27)	144 (21)
Stroke	282 (35)	222 (32)
Peripheral artery disease	318 (40)	255 (37)
Renal arterial stenosis	38 (5)	28 (4)
Atrial fibrillation/flutter	306 (38)	581 (22)
Heart failure	431 (54)	1042 (39)
CKD diagnosis	596 (94)	513 (97)
Glomerular kidney disease	34 (4)	76 (11)
Tubular kidney disease	134 (17)	91 (13)
Membranous nephropathies	4 (1)	16 (2)
Hypertensive kidney disease	440 (55)	465 (68)
CKD unspecified	364 (46)	339 (50)
Cancer	333 (42)	277 (40)
Laboratory measurements^a^		
Systolic BP, mmHg, median (IQR)	130 (120–140)	137 (124–150)
≥140 mmHg, *n* (%)	129 (29)	192 (44)
Haemoglobin, g/dL, median (IQR)	13.1 (11.9–14.4)	12.8 (11.5–14.2)
Potassium, mmol/L, median (IQR)	4.4 (4.1–4.8)	4.4 (4.1–4.8)
eGFR, mL/min/1.73 m^2^, median (IQR)	47 (37–61)	41 (31–55)
45–59 (Stage 3a), *n* (%)	197 (25)	162 (24)
30–44 (Stage 3b), *n* (%)	280 (36)	241 (36)
15–29 (Stage 4), *n* (%)	82 (11)	143 (21)
Creatinine, mg/dL, median (IQR)	1.3 (1.0–1.6)	1.5 (1.2–1.9)
UACR, mg/g, median (IQR)	69.0 (46.0–110.0)	654.5 (360.0–1291.5)
Renoprotective treatment, *n* (%)		
RASi	491 (62)	494 (72)
SGLT2i	0 (0)	0 (0)

a Laboratory measurements represent the last registered value in the year prior to incident CKD.

BP, blood pressure; N/A, not available or not applicable; SD, standard deviation.

### eGFR trajectories and slopes

**Figure 2:**
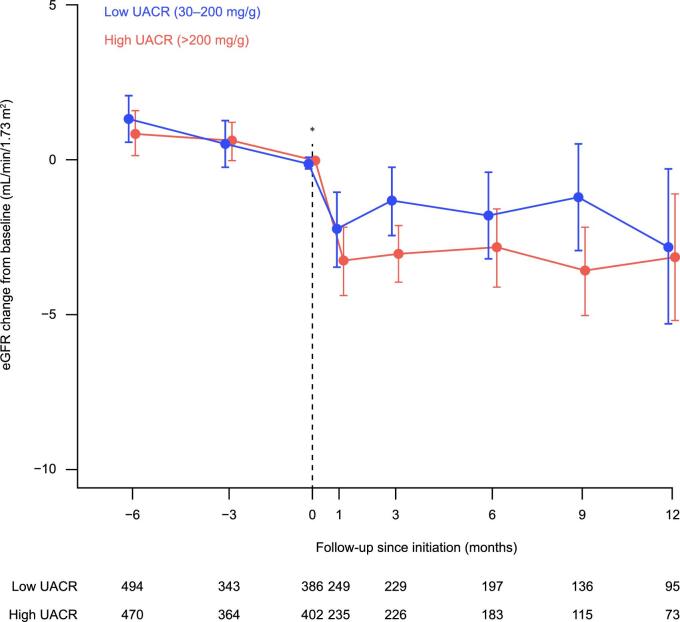
eGFR change from baseline over time following dapagliflozin initiation in patients with CKD without type 2 diabetes in the USA in the years 2021–23 (post-approval). *Initiation of dapagliflozin 10 mg.

In both low and high UACR groups, eGFR showed an acute dip of 3 mL/min/1.73 m^2^ shortly after dapagliflozin initiation (Fig. 2). Change from baseline was similar over time in the low and high UACR groups. The eGFR trajectory did not change when adding patients with normal/mildly elevated UACR to the group with low UACR (Supplementary data, Fig. S1). The trajectory patterns for low and high UACR were similar in patients with type 2 diabetes (Supplementary data, Fig. S2).

**Figure 3:**
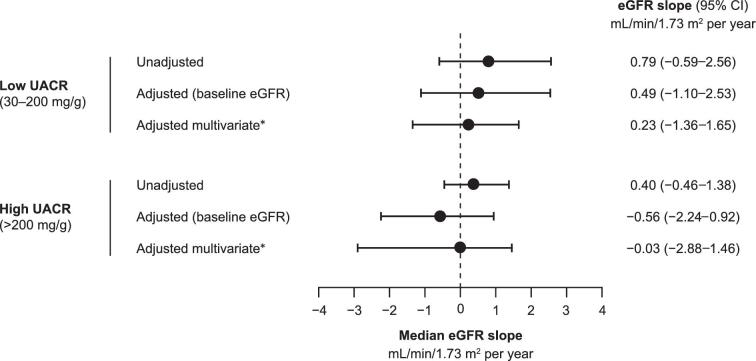
eGFR slopes in patients with CKD without type 2 diabetes initiated with dapagliflozin in the USA in the years 2021–23 (post-approval). *Adjusted for baseline eGFR, age and sex, heart failure and RASi.

The eGFR slope (95% CI) for the low UACR group was flat [0.79 mL/min/1.73 m^2^ per year (–0.59, 2.56)], and similar to patients with high UACR [0.40 mL/min/1.73 m^2^ per year (–0.46, 1.38)] (Fig. 3). The eGFR slope did not change when adding patients with normal/mildly elevated UACR to the group with low UACR (Supplementary data, Fig. S3). Comparable trends were seen for patients with type 2 diabetes with low UACR [0.26 mL/min/1.73 m^2^ per year (–0.33, 1.09)], although a downward slope was observed in patients with type 2 diabetes with high UACR ([–1.45 mL/min/1.73 m^2^ per year (–2.20, –0.71)] (Supplementary data, Fig. S4).

### Cardiorenal and mortality outcomes in low versus high UACR

**Figure 4:**
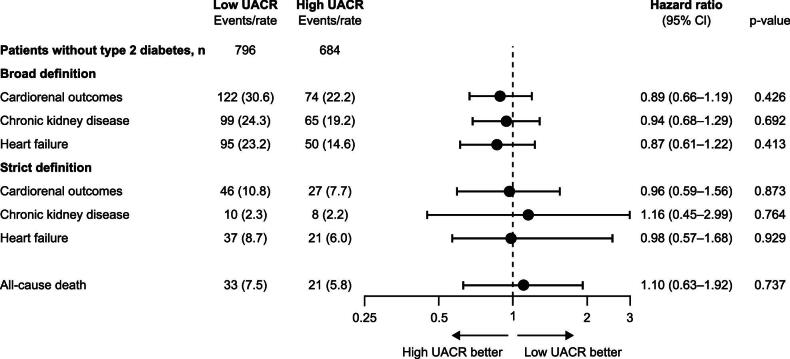
Risk of cardiorenal hospitalizations and all-cause mortality following dapagliflozin initiation in patients with CKD without type 2 diabetes in the USA in the years 2021–23 (post-approval).**Adjusted for age and sex, history of myocardial infarction, stroke, peripheral artery disease, atrial fibrillation, heart failure and RASi.

Risk of hospitalizations for cardiorenal complications and all-cause mortality were similar during follow-up in the low and high UACR groups (Fig. 4), and analogous in patients with type 2 diabetes (Supplementary data, Fig. S5). Using the broad definition, cardiorenal event rates per 100 patient-years were 30.6 and 22.2 in the low and high UACR groups, respectively. The separate components of the combined cardiorenal outcome (CKD and heart failure) had similar event rates in both low (24.3 and 23.2, respectively) and high (19.2 and 14.6, respectively) UACR groups. The adjusted hazard ratio for cardiorenal hospitalization and all-cause mortality in low versus high UACR was 0.89 (95% CI 0.66, 1.19) and 1.10 (95% CI 0.63, 1.92), respectively. Cardiorenal hospitalization results remained close to the line of unity after applying strict criteria [0.96 (95% CI 0.59, 1.56)] (Fig. 4). The risk pattern did not change when adding patients with normal/mildly elevated UACR to the group with low UACR (Supplementary data, Fig. S6). Corresponding observations and cardiorenal risks in low and high UACR groups were analogous in patients with type 2 diabetes [1.12 (95% CI 0.95, 1.32)] (Supplementary data, Fig. S5). In a sensitivity analysis for patients without type 2 diabetes, we added CKD stage to the adjusted model. This did not change the hazard ratios for cardiorenal outcomes [0.89 (95% CI 0.65, 1.20)], CKD [0.92 (95% CI 0.67, 1.28)], heart failure [0.94 (95% CI 0.66, 1.35)] or all-cause mortality [1.18 (95% CI 0.66, 2.10)].

## DISCUSSION

This is one of the first real-world studies of patients with CKD without type 2 diabetes and with low or high UACR initiated on dapagliflozin 10 mg. Following dapagliflozin initiation, changes in kidney function (eGFR trajectories and slopes), time to first cardiorenal hospitalization (CKD or heart failure) and all-cause mortality were similar in patients with low and high UACR. This suggests that the relative effects of dapagliflozin on cardiorenal and mortality outcomes may not vary by UACR status in non-diabetic kidney disease.

There are several important findings in this study. First, patient characteristics were overall similar in the low and high UACR groups. Second, initiation of dapagliflozin was followed by an acute eGFR dip of approximately 3 mL/min/1.73 m^2^ within the first month, with a flat trajectory over the following 12 months. This was similar in both low and high UACR groups, and is aligned with what has been found in clinical trials of SGLT2i in patients with CKD [10]. Third, calculated eGFR slopes were similar in the low and high UACR groups, suggesting an equal degree of kidney protection. Fourth, the risk of cardiorenal complications and all-cause mortality in the high UACR group, where efficacy has been shown in clinical trials [11], was similar to that in the low UACR group, suggesting equal cardiorenal and mortality protection. Fifth, the eGFR trajectories, eGFR slopes and cardiorenal risk findings did not change when adding patients with normal/mildly elevated UACR to the group with low UACR. Sixth, and importantly, the low versus high UACR findings in patients without type 2 diabetes were analogous to patients with type 2 diabetes. In summary, the similarities in eGFR trajectories, eGFR slopes and cardiorenal and mortality risk after initiating dapagliflozin in patients with CKD suggest that the efficacy found in clinical trials is also observed in real-world clinical practice [11, 20, 21], regardless of UACR and diabetes status.

### Patients

Although the patients initiated with dapagliflozin 10 mg in this study were quite similar in age as patients with incident moderate CKD [6, 7] and prevalent CKD [1, 22] in other real-world studies, they had substantially higher comorbid burden (heart failure, atrial fibrillation and atherosclerotic diseases). Furthermore, this burden was slightly higher in patients with low UACR, irrespective of diabetes status. These findings are similar to those of previous reports, where novel drugs like SGLT2i are initially more often prescribed to patients with a higher comorbid burden [23, 24]. Of note, kidney-protective treatment with RASi was lower in patients with low versus high UACR (62% versus 72%, respectively), and also observed in patients with type 2 diabetes. This suggests that patients with CKD and low UACR initiated on dapagliflozin were at a slightly higher baseline risk compared with patients with high UACR.

### eGFR trajectories

The acute eGFR dip following dapagliflozin initiation is analogous to that shown in clinical trials such as DAPA-CKD (Dapagliflozin and Prevention of Adverse Outcomes in Chronic Kidney Disease) [11] and a *post hoc* study of patients without type 2 diabetes (Supplementary data, Fig. S7) [16]. The acute dip in our study was followed by a flat/horizontal chronic eGFR slope, similar in patients with low and high UACR. For patients with low UACR, the eGFR slope was similar irrespective of diabetes status. The dip in eGFR likely reflects a physiological response and the protective mechanism of action of SGLT2i [25]. Interestingly, patients with normal/mildly elevated UACR (0–29 mg/g) showed similar eGFR trajectories and slopes compared with those with low UACR. These data suggest that eGFR-associated kidney protection with dapagliflozin is reproduceable in a real-world setting and extends to patients with CKD without type 2 diabetes, regardless of UACR status.

### Cardiorenal and mortality outcomes

During 12 months of follow-up, cardiorenal and mortality risk developed similarly in the low and high UACR groups. In addition, patients with normal/mildly elevated UACR showed similar cardiorenal and mortality risk development compared with those with low and high UACR. In the much larger sample of patients with diabetes, the same results for low and high UACR were observed. Interestingly, patients initiated on dapagliflozin had similar risk of hospitalization for CKD or heart failure, irrespective of UACR or diabetes status. An important observation, described above, was that the baseline comorbidities (history of heart failure, atrial fibrillation, atherosclerotic cardiovascular disease and time since CKD) at dapagliflozin initiation for the treatment of CKD were similar irrespective of UACR status (normal/mildly elevated, low or high). Hence, the similar baseline risks and observed risk development suggest a consistent risk-reducing clinical benefit of dapagliflozin across different UACR groups. Moreover, the same risk patterns were observed in patients with type 2 diabetes, in whom cardiorenal risk-reducing efficacy has been shown in clinical trials of patients with CKD and normal to high UACR [20].

Interestingly, another study within the OPTIMISE-CKD programme showed a clinically meaningful and significant attenuation on the eGFR slope of 1.09–1.28 mL/1.73 m^2^ per year when comparing dapagliflozin with no treatment in patients without type 2 diabetes with CKD and low UACR [19]. These results were similar to the DAPA-CKD trial results, which showed an eGFR slope attenuation of 0.98 mL/1.73 m^2^ per year when comparing dapagliflozin with placebo [11, 16, 19]. Although our study lacks an untreated control group, it assumes that dapagliflozin's kidney-protective effect in patients with CKD without type 2 diabetes and high UACR from clinical trials translates into a real-world setting. Hence, our study included patients with both high and low UACR and the ability to assess dapagliflozin's effects using a wide range of outcomes. By using two different approaches, we believe that the efforts of the OPTIMISE-CKD study programme are complementary and support the beneficial effects of dapagliflozin in patients with CKD without type 2 diabetes.

In summary, this study shows that dapagliflozin initiation for CKD treatment is followed by similar patterns of kidney protection (eGFR trajectory and slope), and cardiorenal and mortality risks in patients without type 2 diabetes, irrespective of UACR status, supporting its use in a broad CKD patient population.

### Strengths and limitations

Strengths of the study include the large size of the contemporaneous population of patients with CKD, and the consistency of the findings with those observed in clinical trials. Other strengths are the validity of the CKD definition used in the study [1, 26, 27], and the availability of a wide variety of clinical patient characteristics.

Limitations include the recent approval of dapagliflozin for CKD treatment, which limited both the number of patients with CKD without type 2 diabetes and the duration of follow-up time. Because the practical experience is limited by recent approval and low uptake [7], further studies are encouraged. Although the low number of patients might have impacted the statistical strength of the separate analyses, the combined evidence from multiple approaches and additional and identical analyses in a population with type 2 diabetes increases the robustness. The low use of UACR testing in a real-world setting in the USA, as previously reported [6, 7, 22], also limited the number of eligible patients, and introduced a selection bias. Generalization of conclusions to other countries with different healthcare systems or local guidelines might be limited. Diagnosis codes can be misclassified; we therefore considered both any diagnoses and main diagnoses when assessing outcomes. In the USA, complete coverage of mortality data was lacking, which may have affected the competing risk for hospitalization. Safety was not examined, and is out of scope for this study.

## CONCLUSIONS

In this observational study, initiation of dapagliflozin in patients with CKD without type 2 diabetes was followed by multiple similarities in kidney protection and cardiorenal and mortality risks (eGFR trajectories, eGFR slopes, observed cardiorenal hospitalizations and deaths) across all UACR levels. This suggests that the efficacy of dapagliflozin found in clinical trials expands to real-world patients with CKD, regardless of UACR or diabetes status.

## Supplementary Material

sfae100_Supplemental_File

## Data Availability

The data underlying this article will be shared on reasonable request to the corresponding author.
