# Supplementary material for: Dapagliflozin treatment of patients with chronic kidney disease without diabetes across different albuminuria levels (OPTIMISE-CKD)
Source: Clin Kidney J. 2024 Apr 4;17(8):sfae100. doi: 10.1093/ckj/sfae100 (PMC11333959; doi:10.1093/ckj/sfae100)
Supplement: sfae100_Supplemental_File [file sfae100_Supplemental_File.docx]

**SUPPLEMENTAL MATERIAL**

Dapagliflozin treatment of patients with chronic kidney disease without diabetes across different albuminuria levels (OPTIMISE-CKD)

Maria K. Svensson, MD, PhD,^1,2^ Navdeep Tangri, MD, PhD,^3^ Johan Bodegård, MD, PhD,^4^ Samuel Adamsson Eryd, PhD,^4^ Marcus Thuresson, PhD,^5^ Tadashi Sofue, MD, PhD^6^

1. Department of Medical Sciences, Renal Medicine, Uppsala University, Uppsala, Sweden
2. Uppsala Clinical Research Centre, Uppsala, Sweden
3. University of Manitoba Max Rady College of Medicine, Winnipeg, MB, Canada
4. Cardiovascular, Renal and Metabolism Evidence, BioPharmaceuticals Medical, AstraZeneca, Gothenburg, Sweden
5. Statisticon AB, Uppsala, Sweden
6. Department of Cardiorenal and Cerebrovascular Medicine, Kagawa University, Takamatsu, Kagawa, Japan

**CONTENTS**

[SUPPLEMENTAL METHODS 4](#_Toc165647567)

[Data source 4](#_Toc165647568)

[Supplemental Table 1. Inclusion and Exclusion Criteria. 5](#_Toc165647569)

[Supplemental Table 2. Definitions of Comorbidities. 6](#_Toc165647570)

[Supplemental Table 3. Baseline Definitions. 7](#_Toc165647571)

[Supplemental Table 4. Definitions of Drug Treatments. 8](#_Toc165647572)

[Supplemental Table 5. Baseline characteristics. Patients with chronic kidney disease without type 2 diabetes newly initiated on dapagliflozin 10 mg in the United States, years 2021–2023 (post-approval) 9](#_Toc165647573)

[Supplemental Table 6. Baseline characteristics. Patients with chronic kidney disease with and without type 2 diabetes newly initiated on dapagliflozin 10 mg in the United States, years 2021–2023 (post-approval) 11](#_Toc165647574)

[Supplemental Figure 1. eGFR change from baseline over time following dapagliflozin 10 mg initiation in patients with CKD without type 2 diabetes in the United States, years 2021-2023 (post-approval) 13](#_Toc165647575)

[Supplemental Figure 2. eGFR change from baseline over time following dapagliflozin 10 mg initiation in patients with CKD in the United States, years 2021-2023 (post-approval) 14](#_Toc165647576)

[Supplemental Figure 3. eGFR slopes in patients with CKD without type 2 diabetes initiated with dapagliflozin in the USA in the years 2021–23 (post-approval) in which the low UACR group did and did not additionally include patients with normal/mildly elevated UACR (0–29 mg/g). Results in the left-hand panel are reproduced from Figure 3 and are included for reference 15](#_Toc165647577)

[Supplemental Figure 4. eGFR slopes in patients with CKD, with and without type 2 diabetes and initiated with dapagliflozin in the USA in the years 2021–23 (post-approval). Results in the left-hand panel are reproduced from Figure 3 and are included for reference 16](#_Toc165647578)

[Supplemental Figure 5. Risk of cardiorenal hospitalizations following dapagliflozin 10 mg initiation in patients within the United States, years 2021-2023 (post-approval) 17](#_Toc165647579)

[Supplemental Figure 6. Risk of cardiorenal hospitalizations following dapagliflozin 10 mg initiation in patients without type 2 diabetes within the United States, years 2021-2023 (post-approval) 18](#_Toc165647580)

[Supplemental Figure 7. eGFR trajectories in low and high UACR in the DAPA-CKD trial 19](#_Toc165647581)

[REFERENCES 20](#_Toc165647582)

# SUPPLEMENTAL METHODS

## Data source

Optum’s de-identified Clinformatics® Data Mart (CDM) is an administrative claims database for a privately insured population in the United States with commercial or Medicare Advantage coverage. The database covers more than 78 million people with data collected since January 2007 and contains patient-level data from claims submitted for all medical and pharmacy health care services with information related to health care costs and resource utilization. The population is geographically diverse, spanning all 50 states.

As this was an analysis of de-identified claims data compliant with guidance related to the Health Insurance Portability and Accountability Act (HIPAA) privacy rule (1), institutional review board (IRB) approval was not required. Per Title 45 of CFR, Part 46 (2), the administrative claims data analysis in this study was exempt from IRB review as it was a retrospective analysis of existing data (hence no patient intervention or interaction), and no patient-identifiable information was included in the claims dataset.

Supplemental Table 1. Inclusion and Exclusion Criteria.

| **Inclusion Criteria** |
| --- |
| - Age ≥ 18 years as of study index date - With first ever registered laboratory confirmed CKD or CKD diagnosis, defined as at least one of:   - two eGFR measurements ≤60 mL/min/1.73 m^2^ taken ≥90 days apart   - or a first eGFR measurement ≤60 ml/min/1.73 m^2^ followed by a first CKD diagnosis^a^ |
| **Exclusion Criteria** |
| - History of stage 5 CKD, dialysis, type 1 diabetes or gestational diabetes on or before index date |

^a^A full list of diagnosis codes used to identify CKD can be found in Supplemental Table 2.

CKD, chronic kidney disease; eGFR, estimated glomerular filtration rate.

Supplemental Table 2. Definitions of Comorbidities.

| **Disease** | **ICD-10** | **Surgical Code/Drug treatment** |
| --- | --- | --- |
| Heart failure | I50, I11.0, I13.0, I13.2 |  |
| Chronic kidney disease | N17-N19, I12.0-I12.9, I13.1, I13.2, N00-N08, N10-N16 N08.3, E10.2, E11.2, E12.2, E13.2, E14.2, Z49, Z99.2 | Codes for dialysis |
| Cardiovascular diseases |  |  |
| Myocardial infarction | I21-I22, I25.2, I25.6 |  |
| Ischemic heart disease | I21-I22, I25.2, I25.6, I20.9, I20.0 | Codes for revascularizations |
| CABG/PCI |  | Codes for revascularizations |
| Unstable angina | I20.0 |  |
| Angina pectoris | I20.1, I20.8, I20.9, I25.1, I25.5 | Nitrates: C01DA |
| Atrial fibrillation | I48 |  |
| Stroke | I60-I66, G45 |  |
| Hemorrhagic | I60-I62 |  |
| Ischemic | I63 |  |
| Transitory ischemic attack | G45 |  |
| Peripheral artery disease | I70.2, I73.9, I74.2-9 | Code for limb angioplasty |
| Device therapy: Cardioverter and pacemaker |  | Code for device implantation |
| Other diseases |  |  |
| Hyperkalemia | E875 |  |
| Cancer | C00-C99 |  |
| COPD | J44 |  |
| COVID-19 infection | U07.1, U07.2 |  |

CABG, coronary artery bypass graft; COPD, chronic obstructive pulmonary disease; COVID 19, coronavirus disease 2019; ICD, International Classification of Diseases; PCI, percutaneous coronary intervention

Supplemental Table 3. Baseline Definitions.

| **Variable** | **Definition** |
| --- | --- |
| Sex | Male or female |
| Age | Age at index date |
| Index year/month | Year/month of index date |

Supplemental Table 4. Definitions of Drug Treatments.

| **Treatment Category/**  **Class/Medicine** | **ATC Code** | **ATC WHO Code** |
| --- | --- | --- |
| RASi | ACE or ARB or ARNI |  |
| ACEis | C09A, C09B | C09A, C09B |
| ARBs | C09C, C09D (excluding C09DX04) | C09C, C09D excluding treatment name ENTRESTO |
| SGLT-2i | A10BK, A10BD09, A10BD11, A10BD12, A10BD15, A10BD19, A10BD20, A10BD21, A10BD23, A10BD24 | A10P |
| Dapagliflozin | A10BK01, A10BD15, A10BD21 | A10P + treatment name: FORXIGA |
| Antihypertensive treatments |  |  |
| RASi | ACE or ARB |  |
| Calcium channel blockers | C08C |  |
| Thiazides | C03A |  |
| Other treatments |  |  |
| Statins | C10AA | C10A1 |
| Low-dose acetylic salicylic acid | B01AC06, B01AC56, C10BX01, C10BX02, C10BX04, C10BX05, C10BX06, C10BX08, C10BX12, C07FX02, C07FX03, C07FX04, B01AC86, B01AC36, B01AC34 | B01C1 |

ACEi, angiotensin-converting enzyme inhibitor; ARB, angiotensin receptor blocker; ARNI, angiotensin receptor-neprilysin inhibitor; ATC, Anatomical Therapeutic Chemical; DPP-4i, dipeptidyl peptidase-4 inhibitor; GLP-1RA, glucagon-like peptide-1 receptor agonist; MRA, mineralocorticoid receptor antagonist; RASi, renin–angiotensin system inhibitor; SGLT-2i, sodium–glucose cotransporter-2 inhibitor; WHO, World Health Organization

Supplemental Table 5. Baseline characteristics. Patients with chronic kidney disease without type 2 diabetes newly initiated on dapagliflozin 10 mg in the United States, years 2021–2023 (post-approval)

|  | **Patients without type 2 diabetes** | | |
| --- | --- | --- | --- |
|  | **Normal/mildly elevated**  0-29 mg/g | **Low UACR**  30-200 mg/g | **High UACR**  >200 mg/g |
| Number of patients, n (%) | 1549 (51) | 796 (26) | 684 (23) |
| Age, years, mean (SD) | 75 (8) | 75 (8) | 74 (9) |
| Female, n (%) | 724 (47) | 336 (42) | 264 (39) |
| Days since 1st CKD diagnosis, median (IQR) | 1284 (554-2073) | 1347 (618-2024) | 1169 (538-2067) |
| **Comorbidities** |  |  |  |
| Atherosclerotic cardiovascular disease |  |  |  |
| Myocardial infarction, n (%) | 411 (27) | 215 (27) | 144 (21) |
| Stroke, n (%) | 534 (34) | 282 (35) | 222 (32) |
| Peripheral artery disease, n (%) | 593 (38) | 318 (40) | 255 (37) |
| Atrial fibrillation/flutter, n (%) | 509 (33) | 306 (38) | 193 (28) |
| HF, n (%) | 817 (53) | 431 (54) | 269 (39) |
| CKD diagnosis, n (%) | 1435 (93) | 750 (94) | 665 (97) |
| Cancer, n (%) | 640 (41) | 333 (42) | 277 (40) |
| **Laboratory measurements^a^** |  |  |  |
| Systolic BP, mmHg, median (IQR) | 128 (118-140) | 130 (120-140) | 137 (124-150) |
| ≥ 140 mmHg, n (%) | 220 (26) | 129 (29) | 192 (44) |
| Hemoglobin, g/dL, median (IQR) | 13.1 (12.0-14.4) | 13.1 (11.9-14.4) | 12.8 (11.5-14.2) |
| Potassium, mmol/L, median (IQR) | 4.4 (4.1-4.7) | 4.4 (4.1-4.8) | 4.4 (4.1-4.8) |
| eGFR, mL/min/1.73 m^2^, median (IQR) | 50 (40-63) | 47 (37-61) | 41 (31-55) |
| 45–59 (Stage 3a), n (%) | 534 (35) | 197 (25) | 162 (24) |
| 30–44 (Stage 3b), n (%) | 453 (30) | 280 (36) | 241 (36) |
| 15–29 (Stage 4), n (%) | 111 (7) | 82 (11) | 143 (21) |
| Creatinine, mg/dL, median (IQR) | 1.2 (1.0-1.5) | 1.3 (1.0-1.6) | 1.5 (1.2-1.9) |
| UACR, mg/g, median (IQR) | 9.0 (5.0-16.0) | 69.0 (46.0-110.0) | 654.5 (360.0-1291.5) |
| **Renoprotective treatment** |  |  |  |
| RASi, n (%) | 959 (62) | 491 (62) | 494 (72) |
| SGLT2i, n (%) | 0 (0) | 0 (0) | 0 (0) |

^a^Laboratory measurements represent the last registered value in the year prior to incident CKD. BP, blood pressure; CKD, chronic kidney disease; eGFR, estimated glomerular filtration rate; HF, heart failure; N/A, not available or not applicable; RASi, renin–angiotensin system inhibitor; SGLT-2i, sodium–glucose cotransporter-2 inhibitor; UACR, urine albumin-creatinine ratio.

Supplemental Table 6. Baseline characteristics. Patients with chronic kidney disease with and without type 2 diabetes newly initiated on dapagliflozin 10 mg in the United States, years 2021–2023 (post-approval)

|  | **Patients without type 2 diabetes** | | **Patients with type 2 diabetes** | |
| --- | --- | --- | --- | --- |
|  | **Low UACR**  30-200 mg/g | **High UACR**  >200 mg/g | **Low UACR**  30-200 mg/g | **High UACR**  >200 mg/g |
| Number of patients, n (%) | 796 (26) | 684 (23) | 2411 (31) | 1983 (26) |
| Age, years, mean (SD) | 75 (8) | 74 (9) | 74 (8) | 72 (8) |
| Female, n (%) | 336 (42) | 264 (39) | 1079 (45) | 797 (40) |
| Days since 1st CKD diagnosis, median (IQR) | 1347 (618-2024) | 1169 (538-2067) | 1064 (464-1870) | 1100 (481-1931) |
| **Comorbidities** |  |  |  |  |
| ASCVD, n (%) |  |  |  |  |
| Myocardial infarction, n (%) | 215 (27) | 144 (21) | 456 (19) | 399 (20) |
| Stroke, n (%) | 282 (35) | 222 (32) | 748 (31) | 602 (30) |
| Peripheral artery disease, n (%) | 318 (40) | 255 (37) | 826 (34) | 712 (36) |
| Renal arterial stenosis, n (%) | 38 (5) | 28 (4) | 44 (2) | 52 (3) |
| Atrial fibrillation/flutter, n (%) | 306 (38) | 193 (28) | 595 (25) | 388 (20) |
| HF, n (%) | 431 (54) | 269 (39) | 927 (38) | 773 (39) |
| CKD diagnosis, n (%) | 750 (94) | 665 (97) | 2241 (93) | 1921 (97) |
| Glomerular kidney disease | 34 (4) | 76 (11) | 42 (2) | 113 (6) |
| Tubular kidney disease | 134 (17) | 91 (13) | 330 (14) | 228 (11) |
| Membranous nephropathies | 4 (1) | 16 (2) | 3 (0) | 10 (1) |
| Hypertensive kidney disease | 440 (55) | 465 (68) | 1204 (50) | 1241 (63) |
| CKD unspecified | 364 (46) | 339 (50) | 854 (35) | 932 (47) |
| Cancer, n (%) | 333 (42) | 277 (40) | 828 (34) | 571 (29) |
| **Laboratory measurements^a^** |  |  |  |  |
| Systolic BP, mmHg, median (IQR) | 130 (120-140) | 137 (124-150) | 132 (122-145) | 136 (126-149) |
| ≥ 140 mmHg, n (%) | 129 (29) | 192 (44) | 444 (34) | 480 (42) |
| Hemoglobin, g/dL, median (IQR) | 13.1 (11.9-14.4) | 12.8 (11.5-14.2) | 12.9 (11.8-14.2) | 12.6 (11.3-13.9) |
| Potassium, mmol/L, median (IQR) | 4.4 (4.1-4.8) | 4.4 (4.1-4.8) | 4.5 (4.2-4.8) | 4.5 (4.2-4.9) |
| eGFR, mL/min/1.73 m^2^, median (IQR) | 47 (37-61) | 41 (31-55) | 50 (38-66) | 44 (34-58) |
| 45–59 (Stage 3a), n (%) | 197 (25) | 162 (24) | 655 (28) | 483 (25) |
| 30–44 (Stage 3b), n (%) | 280 (36) | 241 (36) | 701 (30) | 687 (36) |
| 15–29 (Stage 4), n (%) | 82 (11) | 143 (21) | 255 (11) | 325 (17) |
| Creatinine, mg/dL, median (IQR) | 1.3 (1.0-1.6) | 1.5 (1.2-1.9) | 1.2 (1.0-1.6) | 1.4 (1.1-1.8) |
| UACR, mg/g, median (IQR) | 69.0 (46.0-110.0) | 654.5 (360.0-1291.5) | 70.0 (46.0-111.0) | 623.0 (332.0-1372.0) |
| **Renoprotective treatment** |  |  |  |  |
| RASi, n (%) | 491 (62) | 494 (72) | 1860 (77) | 1585 (80) |
| SGLT2i, n (%) | 0 (0) | 0 (0) | 0 (0) | 0 (0) |

^a^Laboratory measurements represent the last registered value in the year prior to incident CKD. BP, blood pressure; CKD, chronic kidney disease; eGFR, estimated glomerular filtration rate; HF, heart failure; N/A, not available or not applicable; RASi, renin–angiotensin system inhibitor; SGLT-2i, sodium–glucose cotransporter-2 inhibitor; UACR, urine albumin-creatinine ratio.

Supplemental Figure 1. eGFR change from baseline over time following dapagliflozin 10 mg initiation in patients with CKD without type 2 diabetes in the United States, years 2021-2023 (post-approval)


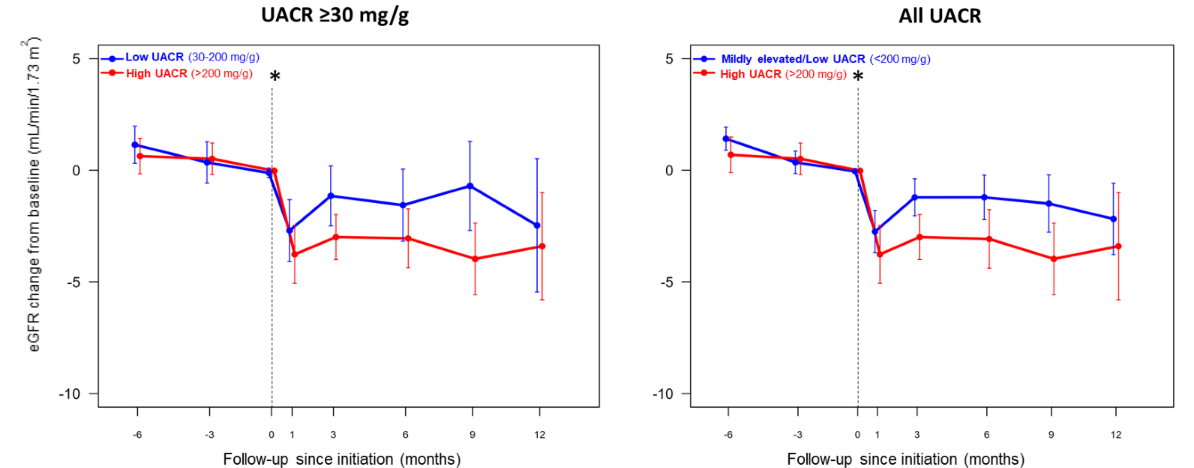


*Initiation of dapagliflozin 10 mg

CKD, chronic kidney disease; eGFR, estimated glomerular filtration rate; UACR, urine albumin-creatinine ratio

Supplemental Figure 2. eGFR change from baseline over time following dapagliflozin 10 mg initiation in patients with CKD in the United States, years 2021-2023 (post-approval)


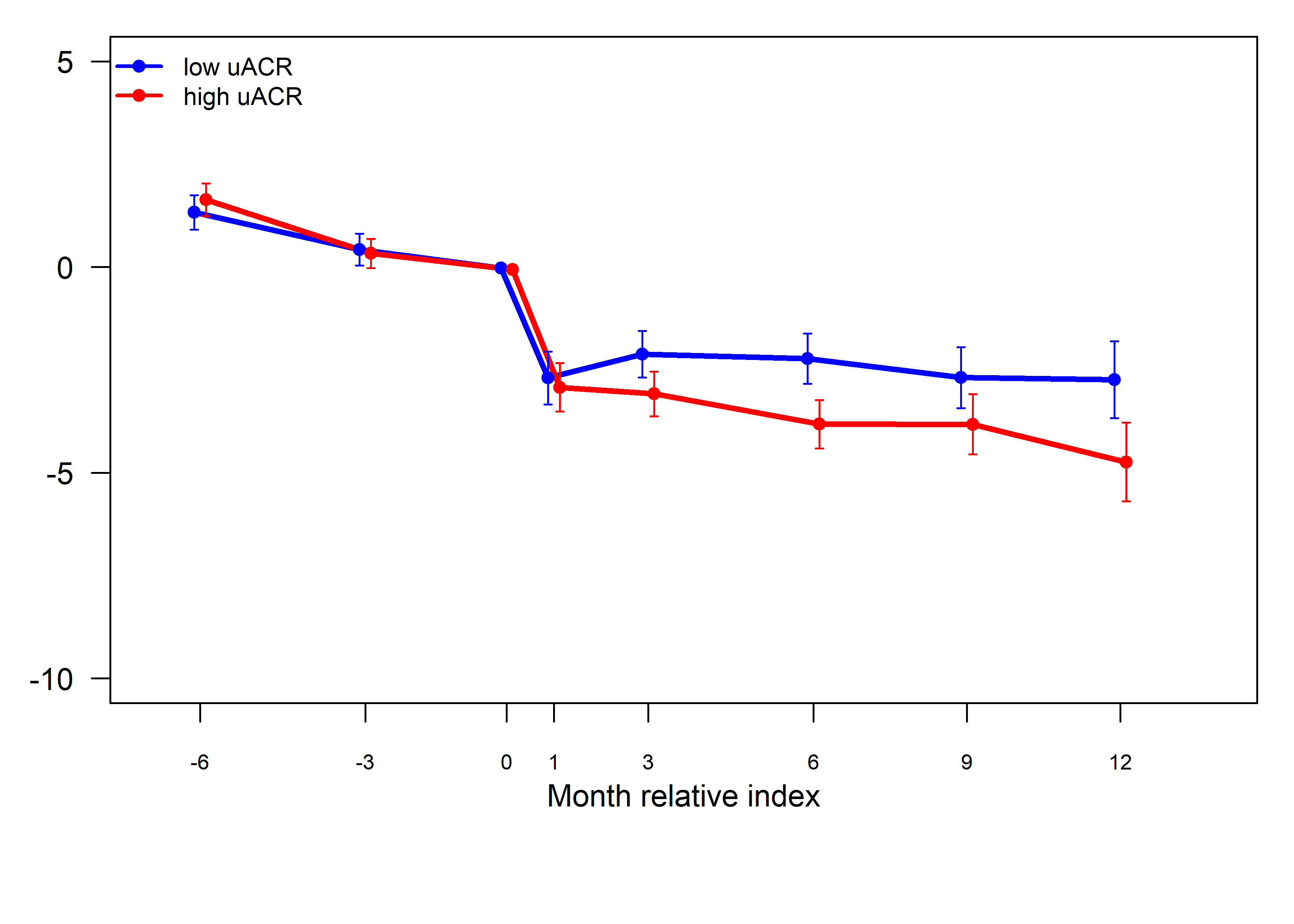


**Patients with type 2 diabetes**

**Low UACR (**30-200 mg/g)

**High UACR** (>200 mg/g)


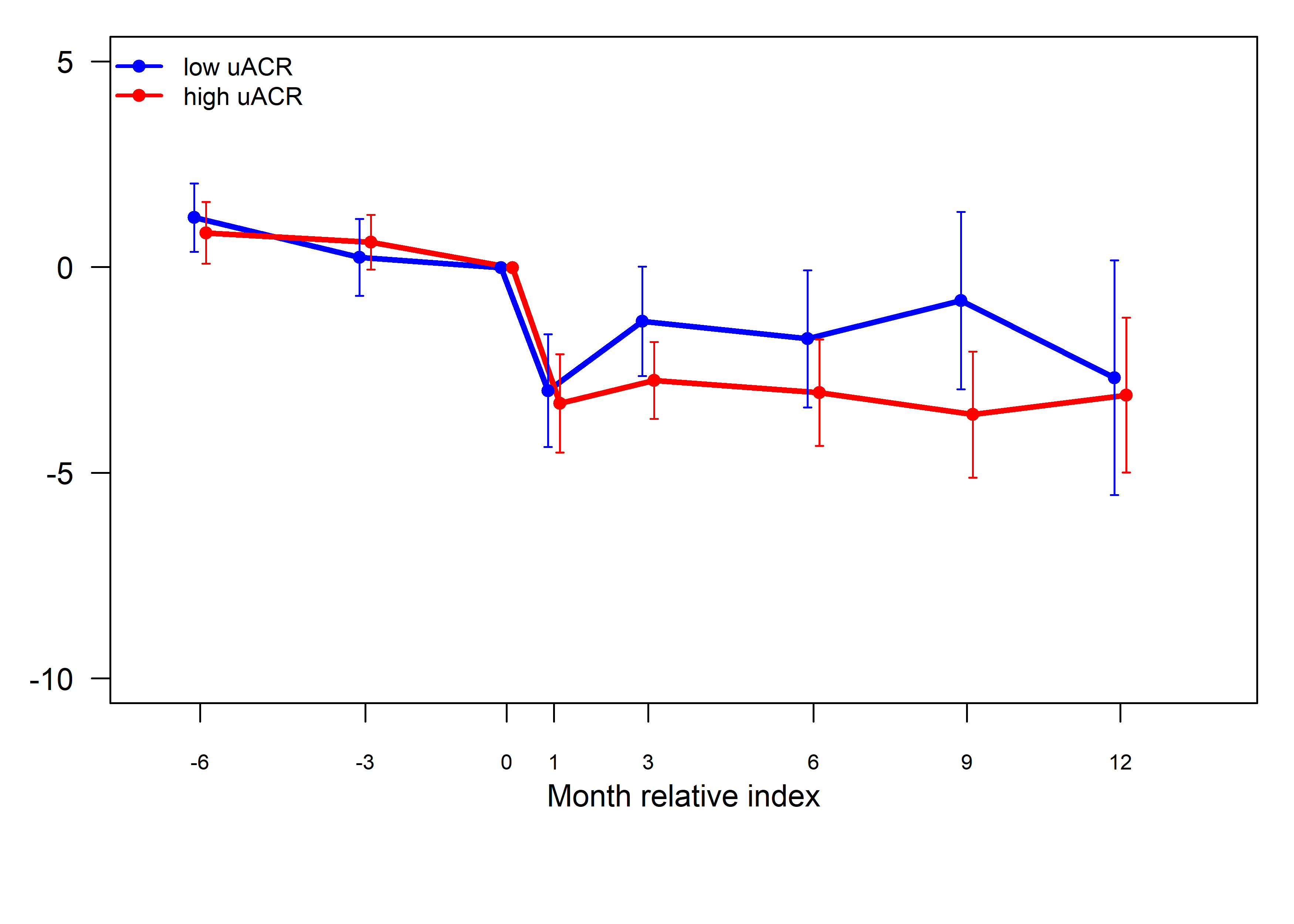


**Patients without type 2 diabetes**

**Low UACR (**30-200 mg/g)

**High UACR** (>200 mg/g)

*

*

*Initiation of dapagliflozin 10 mg

Supplemental Figure 3. eGFR slopes in patients with CKD without type 2 diabetes initiated with dapagliflozin in the USA in the years 2021–23 (post-approval) in which the low UACR group did and did not additionally include patients with normal/mildly elevated UACR (0–29 mg/g). Results in the left-hand panel are reproduced from Figure 3 and are included for reference


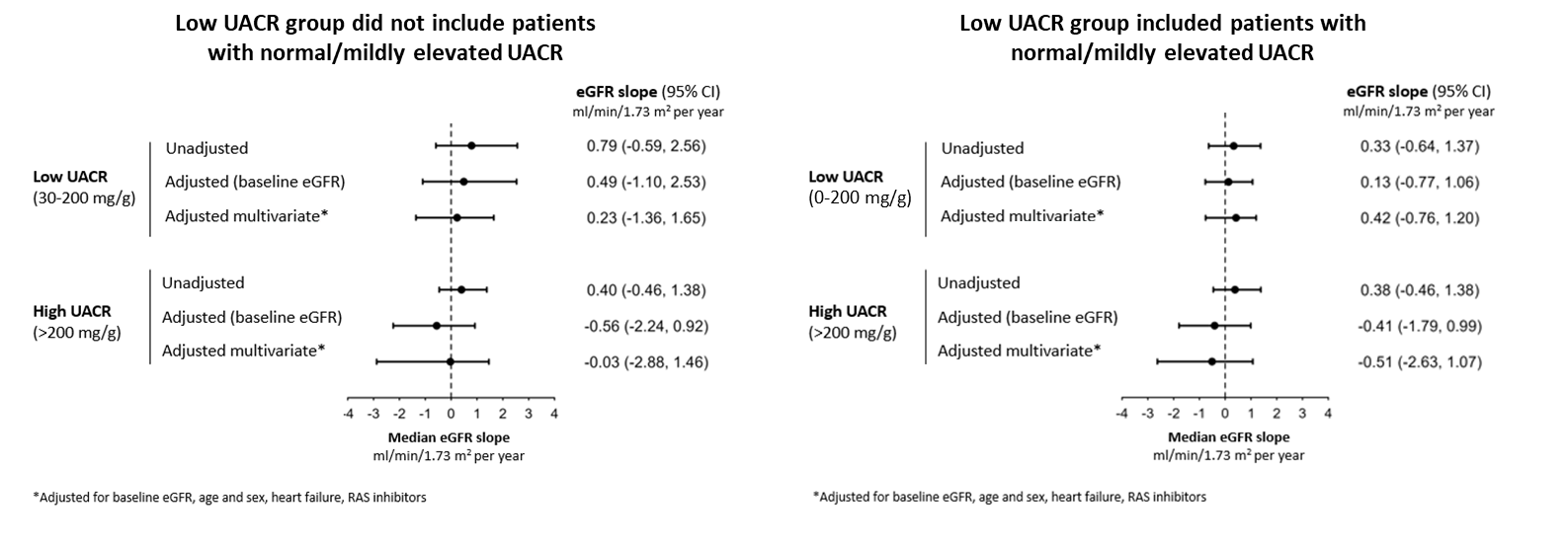


**Supplemental Figure 4.** eGFR slopes in patients with CKD, with and without type 2 diabetes and initiated with dapagliflozin in the USA in the years 2021–23 (post-approval). Results in the left-hand panel are reproduced from Figure 3 and are included for reference


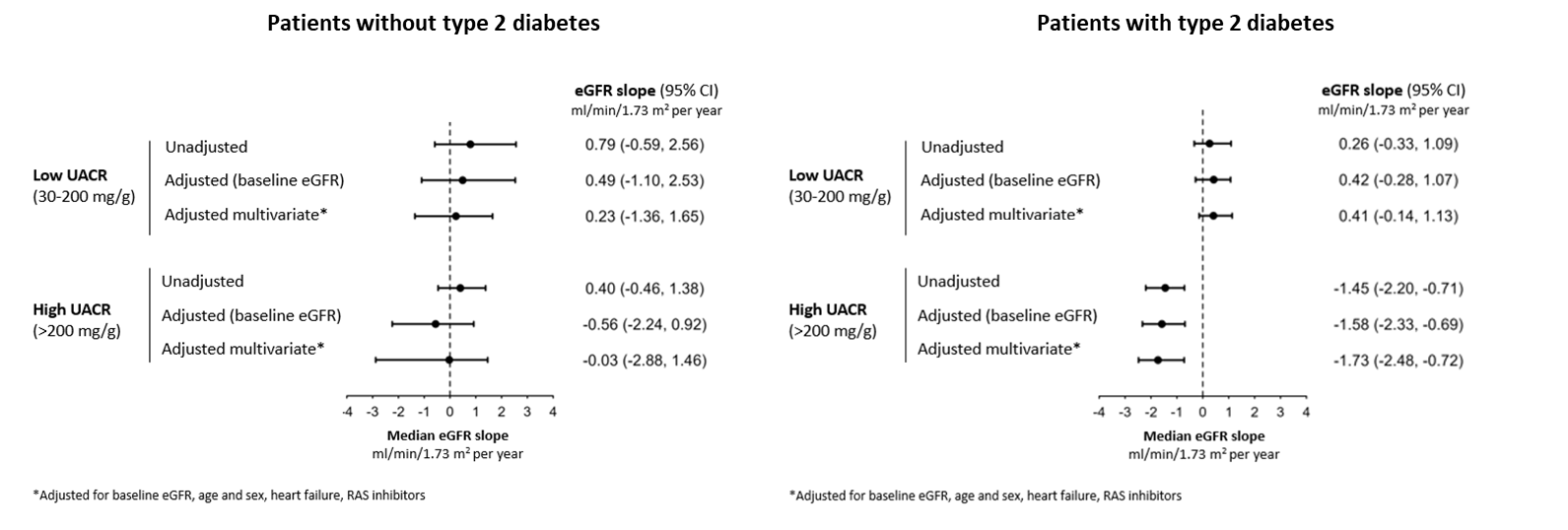


Supplemental Figure 5. Risk of cardiorenal hospitalizations following dapagliflozin 10 mg initiation in patients within the United States, years 2021-2023 (post-approval)


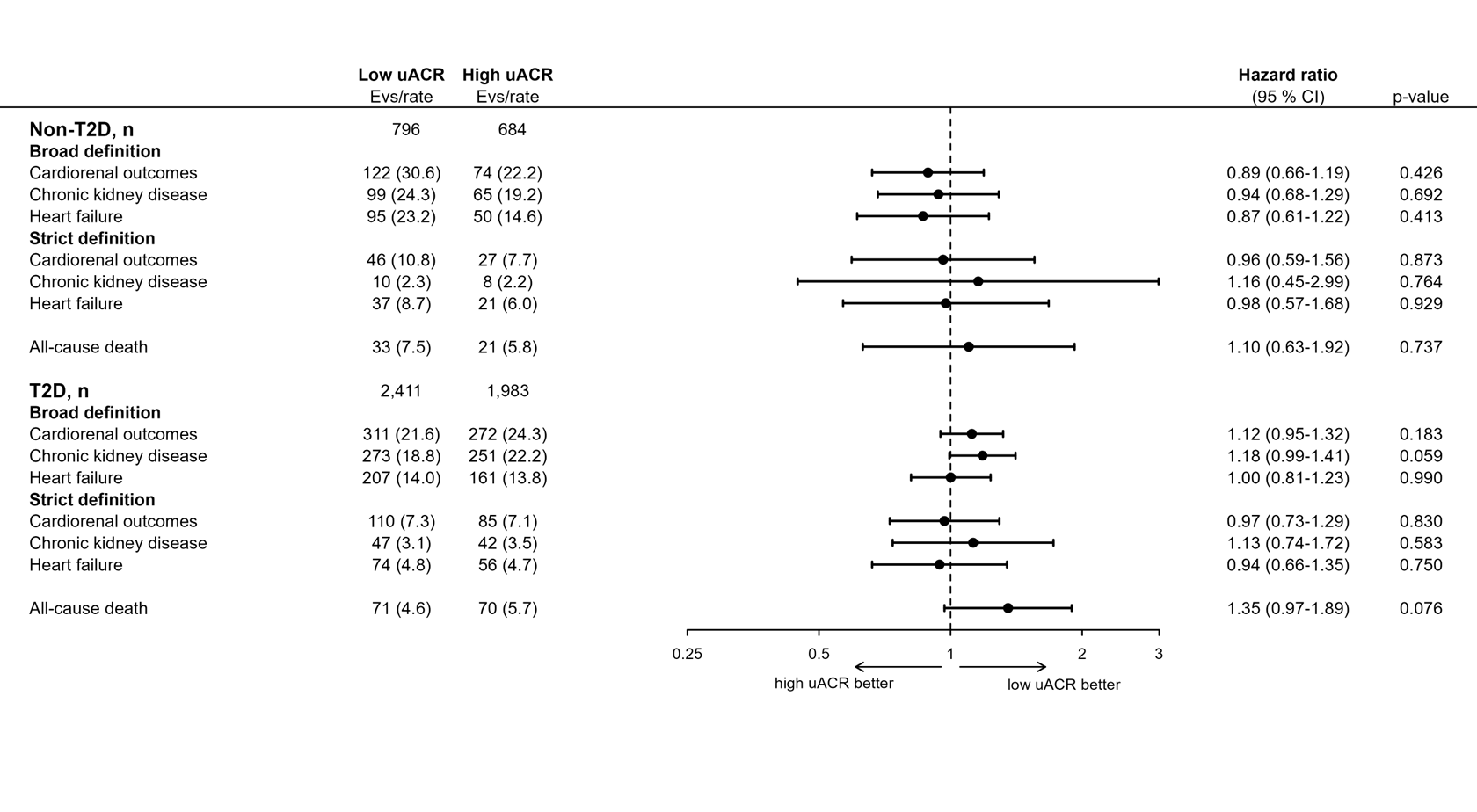


*Adjusted for age and sex, history of myocardial infarction, stroke, peripheral artery disease, atrial fibrillation, heart failure, RAS inhibitors

Supplemental Figure 6. Risk of cardiorenal hospitalizations following dapagliflozin 10 mg initiation in patients without type 2 diabetes within the United States, years 2021-2023 (post-approval)


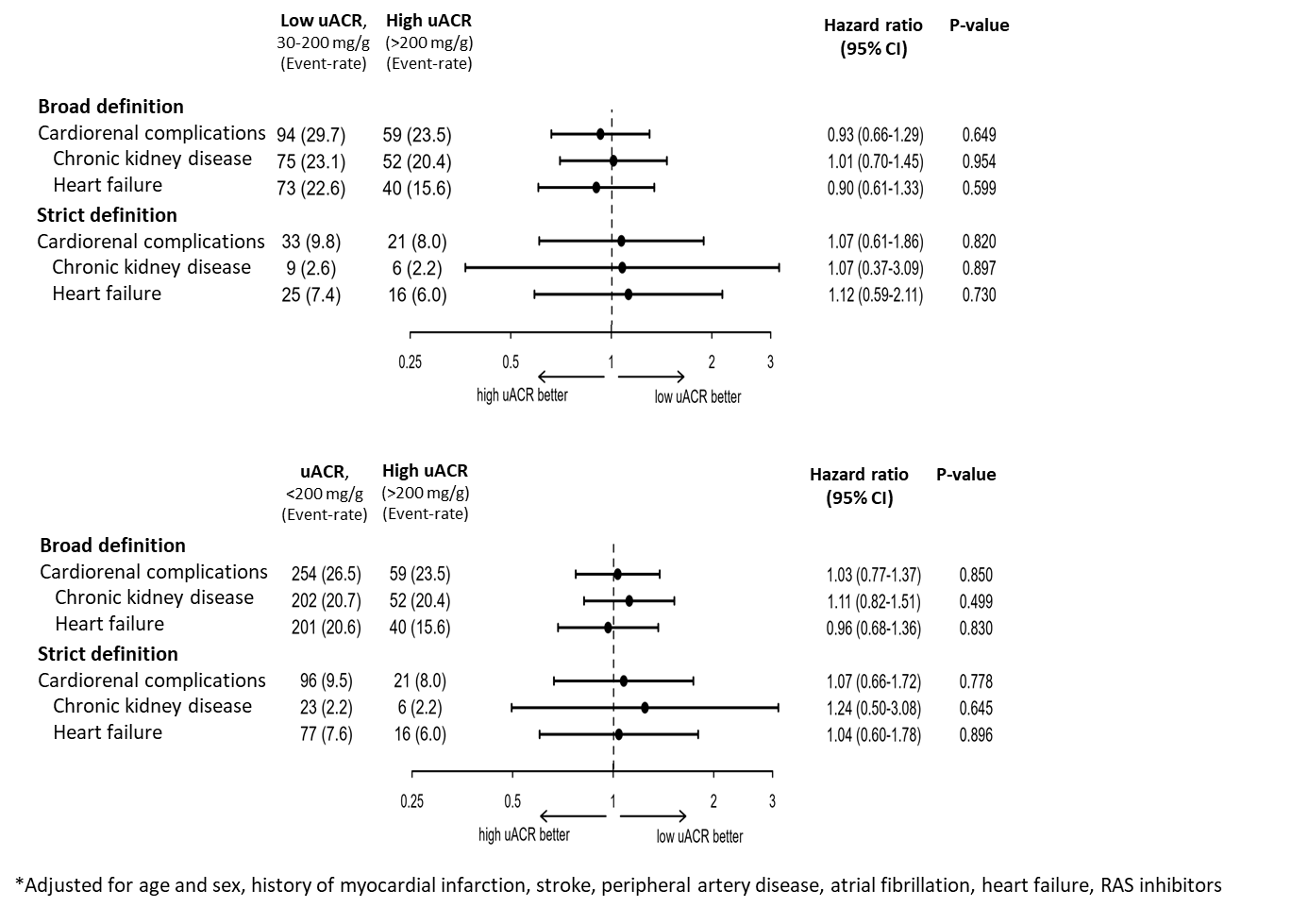


Supplemental Figure 7. eGFR trajectories in low and high UACR in the DAPA-CKD trial


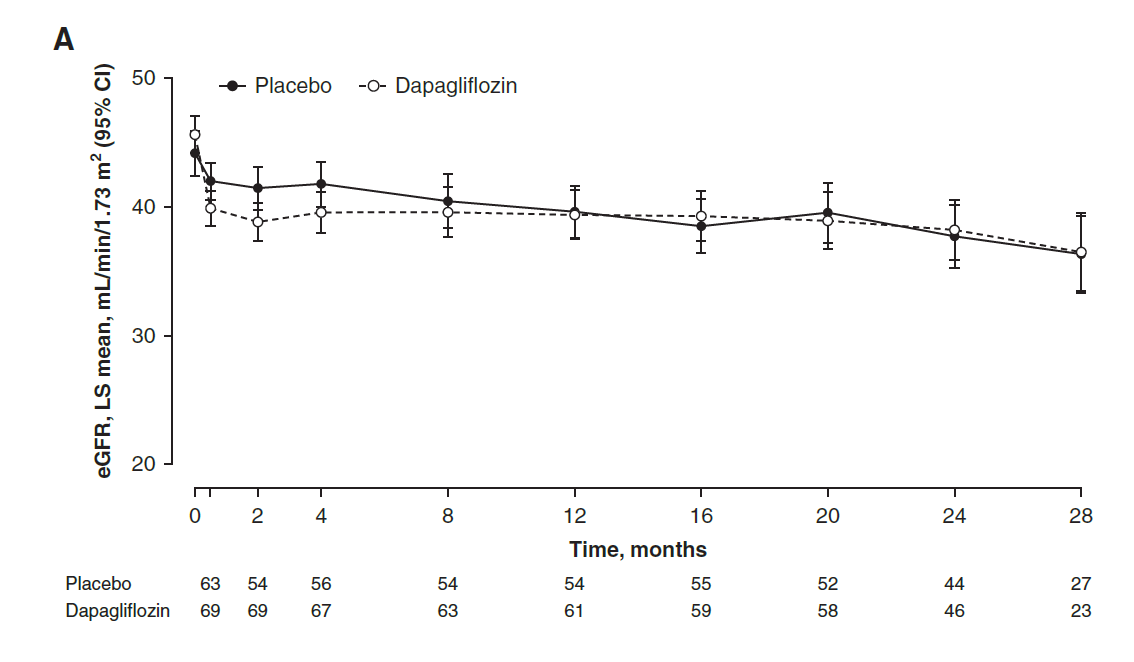


**Low uACR, n=24 (**30-300 mg/g)

**High uACR, n=1262** (>300 mg/g)


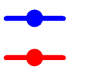


Adapted with permission from Wolters Kluwer Health, Inc.: Heerspink HJL, Chertow GM, Jongs N, Correa-Rotter R, Rossing P, Sjostrom CD, et al. Effects of Dapagliflozin in People without Diabetes and with Microalbuminuria. Clin J Am Soc Nephrol. 2022;17(11):1665-8. https://www.ncbi.nlm.nih.gov/pmc/articles/PMC9718033/ (3)

# REFERENCES

1. US Department of Health and Human Services Guidance Regarding Methods for De-identification of Protected Health Information in Accordance with the Health Insurance Portability and Accountability Act (HIPAA) Privacy Rule: US Department of Health and Human Services; 2022 [Available from: <https://www.hhs.gov/hipaa/for-professionals/privacy/special-topics/de-identification/index.html>.

2. US Department of Health and Human Services 45 CFR 46: US Department of Health and Human Services; 2021 [Available from: [www.hhs.gov/ohrp/humansubjects/guidance/45cfr46.html#46.101](file://10.200.64.12/Projects/AstraZeneca/AZRD%20RWE%20Diabetes/AZRD618%20-%20OPTIMISE-CKD%20uACR%20manuscript/Manuscript/www.hhs.gov/ohrp/humansubjects/guidance/45cfr46.html#46.101).

3. Heerspink HJL, Chertow GM, Jongs N, Correa-Rotter R, Rossing P, Sjostrom CD, et al. Effects of Dapagliflozin in People without Diabetes and with Microalbuminuria. Clin J Am Soc Nephrol. 2022;17(11):1665-8.
